# How to integrate physical activity and exercise approaches into inpatient treatment for eating disorders: fifteen years of clinical experience and research

**DOI:** 10.1186/s40337-018-0203-5

**Published:** 2018-09-25

**Authors:** Marit Danielsen, Øyvind Rø, Sigrid Bjørnelv

**Affiliations:** 1 Specialist Eating Disorder Unit, Department of Psychiatry, Levanger Hospital, Hospital Trust Nord-Trøndelag, NO-7600 Levanger, Norway; 2 Department of Mental Health, Faculty of Medicine and Health Sciences, Norwegian University of Science and Technology, Trondheim, Norway; 3 Regional Eating Disorder Service, Division of Mental Health and Addiction, Oslo University Hospital, Oslo, Norway; 4 Division of Mental Health and Addiction, Institute of Clinical Medicine, University of Oslo, Oslo, Norway

**Keywords:** Eating disorders, Inpatient treatment, Physical activity, Exercise, Clinical experience

## Abstract

**Background:**

The importance of physical activity and exercise among patients with eating disorders (EDs) is acknowledged among clinicians and researchers. The lack of clinical guidelines, the differing attitudes towards exercise approaches in treatment, and the lack of specialized competence all influence the management of ED symptoms in specialist ED treatment units. The purpose of the study was to examine 15 years of clinical experience with exercise approaches as an integrated part our inpatients treatment program.

**Methods:**

From January 2003 to December 2017, 244 patients were admitted to a specialist ED unit in Norway. The treatment program at the ED unit is multidisciplinary. It is based on psychodynamic theory, and designed to enhance patients’ recovery and to enable them to adopt a lifestyle that is as healthy as possible. The authors describe the clinical management of patients with reference to practical examples and a case example.

**Recommendations and experience:**

The treatment for exercise is not manualized, but adjusted to the specific symptoms and needs of individual ED patients. The treatment approaches to exercise are part of the body-oriented treatment at the Specialist eating disorder unit (Regionalt kompetansesenter for spiseforstyrrelser (RKSF)), and the therapy addresses the entire body and the relation between the body, emotions, and the patient’s social situation. It covers a chain of approaches from admission to discharge, from rest and relaxation to regular exercise groups.

**Conclusions:**

Our experience and recommendations support earlier proposals for treatment approaches to exercise and exercise-related issues as a beneficial supplement to the treatment of ED inpatients. We have not experienced any adverse influences on patients’ recovery processes, such as their rate of weight gain. Our intention is that this paper will be a contribution to the field of ED, the integration of exercise approaches in the inpatient treatment of ED and development of clinical guidelines.

## Background

The importance of physical activity and exercise among patients with eating disorders (EDs) has been acknowledged among clinicians and researchers [1], and research has shown associations between physical activity and exercise and the course of the illness [2, 3]. However, the lack of guidelines for managing physical activity and exercise, the differing attitudes towards such approaches in treatment, and the lack of specialized competence all influence the management of ED symptoms in specialist ED treatment units [4].

Physical activity and exercise have been characterized as harmful in the treatment of EDs, but when performed in a therapeutic setting they can be a beneficial supplement to such treatment [2, 4, 5]. Physical activity and exercise have been defined as a harmful strategy for losing weight and as a compensatory behavior in the ED diagnostic criteria [6], as well as important for the maintenance of ED symptoms [7]. Hence, it is important to consider different motives for doing physical activity and exercise, such as exercise for weight, shape, and appearance reasons [8, 9], exercise for regulation of emotions, and compulsivity, rigidity, and negative consequences if physical activity and/or exercise are restricted or stopped [10]. [In the case of persons with EDs, it is important to be aware also of the kind of compulsive activity they do, which may not necessarily be recognized as exercise, such as whether they are always restless, standing, walking, or rejecting all forms of relaxation [11]. Additionally, researchers have emphasized that it is more important to address qualitative elements in ED patients’ exercise, such as attitudes and thoughts, rather than the amount of exercise, they do [12]. However, some researchers have suggested that participation in supervised exercise interventions may have a positive influence on the treatment of ED patients. There are indications of reduced ED symptoms [13], enhanced quality of life, psychological well-being, and patient satisfaction [2, 3] as a result of such interventions. Furthermore, targeted exercise interventions do not have adverse impact on weight gain for anorexia nervosa (AN) patients [2, 3, 5, 14]. As early as 1994, Beumont and colleagues proposed a supervised exercise program for ED patients would be a beneficial supplement to existing treatment [15]. A study by Bratland-Sanda and colleagues has shown that such approaches have been more likely to be included in the treatment of a binge eating disorder (BED) than the treatment of AN and bulimia nervosa (BN) [4]. However, the authors of a more recent study have reported increasing agreement among clinical experts regarding the importance of physical activity and exercise for AN patients [1]. They confirmed that the experts’ preferred term was *compulsive exercise*, and that positive attitudes towards exercise approaches in the treatment of AN were emphasized. Hereafter in this paper, we use the term *exercise*, but it should be borne in mind that the term covers all types and levels of physical activity.

Assessment of exercise symptoms (qualitative and quantitative), and adjusted treatment approaches have been actively integrated in our specialist ED unit for 15 years. In order to offer treatment in accordance with the symptoms of EDs, we decided to integrate exercise approaches into the treatment program. Arguments in favor of this choice in many ways corresponded to the arguments presented by Beumont and colleagues in 1994 [15]. They pointed out that if negative exercise is not addressed during treatment, patients may be discharged without receiving help to understand both their symptoms and how to perform their activities in a healthy way.

The purpose of this paper is to describe 15 years of clinical experience with exercise approaches as an integrated part our inpatients treatment program. We describe the clinical management of patients with reference to practical examples and a case example, and present our recommendations based on the results of the treatment program.

## Methods

### Participants and procedure

Between January 2003 and December 2017, 244 patients were admitted to our specialist ED unit (Regionalt kompetansesenter for spiseforstyrrelser (RKSF)), which serves the Central Norway Regional Health Authority. It has eight beds for treating patients aged 18 years or over, but patients as young as 16 years can be referred to the unit. Descriptive information regarding age at diagnosis, admission time, and body mass index (BMI) is listed in Table 1. Indications for admission were symptom severity and lack of satisfactory improvement achieved by outpatient treatment. With one exception, all admissions were voluntary.

**Table 1:** Descriptive information: age, admission time, and BMI in the whole sample and in diagnostic groups

	Whole sample *N* = 244	Anorexia nervosa (F50.0) *n* = 140	Atypical anorexia nervosa (F50.1) *n* = 33	Bulimia nervosa (F50.2 and F50.3) *n* = 56	Unspecified eating disorder (F50.9) *n* = 15
	Mean (SD)	Mean (SD)	Mean (SD)	Mean (SD)	Mean (SD)
Age (years) Range	21.6 (4.4) 16.0–46.7	21.0 (4.1) 16.0–46.5	23.0 (6.3) 17.1–46.7	22.2 (3.3) 16.7–34.8	22.0 (5.0) 18.3–38.3
Admission time (days) Range	121 (72) 1–358	141 (76) 1–358	96 (57) 7–251	101 (52) 2–207	81 (60) 25–231
	*n* = 243		*n* = 32		
BMI^a^ Range	18.1 (6.0) 11.1–62.1	15.0 (1.6) 11.1–18.2	19.4 (1.1) 17.5–21.3	21.8 (3.6) 16.5–32.3	33.6 (13.6) 21.8–62.1

^a^Reduced n due to missing BMI data for one patient at admission

Our guidelines and practical management of exercise in treatment have been developed through quality assurance work, and are based on of clinical experience, observations, and research. Compulsive attitudes towards and thoughts about exercise and exercise behavior were regularly reported among our patients. As a part of our clinical examination, we included assessments relating to exercise as early as in 2003. We paid special attention to the patients’ attitudes towards and thoughts about exercise. However, we also considered their previous exercise experience, including the type, frequency, intensity, and duration of the exercise. This information was (and still is) integrated in the planning and evaluation of each patient’s treatment.

### Treatment program

The treatment program is multidisciplinary. It is based on psychodynamic theory, but includes elements of cognitive behavioral therapy and motivational interviewing. The program has been described in more detail in an earlier publication [16], and is therefore only briefly summarized in this section. The treatment provided at the unit is designed to enhance patients’ recovery and to encourage them to adopt a lifestyle that is as healthy as possible. The main goals are to establish normal eating behavior, address physical complications, and target psychological issues. The majority of the staff are specialists in their respective professions. Each patient is allocated a primary care team, consisting of two nurses, a psychologist or a psychiatrist, and a physiotherapist. However, the psychiatrists are responsible for continuous medical monitoring of all patients.

There are three stages in the treatment program. After an introductory week, patients who decide to stay sign a treatment agreement approving the terms of their treatment. The unit has an obligatory weekly schedule of group therapy and individual therapy sessions. All patients enter the regular treatment program in Stage 1, in which the focus is primarily directed towards meeting their basic needs through rest, regular meals, and an adequate circadian rhythm. In a meeting 4 weeks after admission, the primary care team and the multidisciplinary staff in the unit jointly discuss all assessments and observations. During the meeting, each patient’s treatment targets, admission time, and adjustments to the treatment are finalized. For underweight patients, admission time is estimated as the number of weeks deemed necessary for them to reach the target BMI in the unit (BMI 20). Underweight patients have to achieve an average weight gain of 0.7 kg per week until their target BMI is reached. The criteria for transitioning from Stage 1 to Stage 2 are based on factors such as the patient’s responsibility for meeting their own needs, their ability to follow the routines relating to meals and appointments, and their ability of participation in treatment approaches. The minimum time spent in Stage 1 is 2 weeks, but underweight patients also have to achieve a BMI of approximately 17 as a transition criterion.

Stage 2 is defined as the active treatment phase. It is not restricted to a specific number of weeks. Instead, the focus is on reaching treatment goals and target weight. Stage 3 starts 6 week before discharge and is a weight stabilization period, but attention is increasingly directed towards life outside the unit. If the treatment agreement is broken during the treatment stages, patients may be given 1 week to adjust to their plan. In the absence of planned progress, the patient may be required to leave the treatment for 1 week. After that week, the patient has to renew their commitment to the treatment and both they and their primary care team discussed whether or how the treatment should be continued.

## Experience and recommendations

In our unit, the treatment approaches to exercise are part of the body-oriented treatment in the RKSF, and the therapy addresses the entire body and the relation between the patient’s body, emotions, and social situation [15]. In order to address the complexity of exercise symptoms in the patients, we have established chains of approaches from admission to discharge, and from rest and relaxation to regular exercise groups. These interventions are modified to complement each patient’s treatment plan and individual needs, and we perceive exercise as a complex phenomenon.

Our guidelines are based on clinical experience, research, and quality assurance work. In the following, we describe approaches and issues relating to exercise that should be considered in treatment of EDs, and present some elements from the practical management of ED patients though a case example. This is not the story of one particular patient, but rather serves to shed light on commonly experienced challenges in the course of underweight patients in inpatient treatment. The “patient’s” pseudonym is “Eva.”

### General principles

The treatment for exercise is not manualized, but is adjusted to the specific symptoms and needs of ED patients, and is based on Norwegian Directorate of Health’s guidelines for physical activity in treatment and prevention [17]. It is important to establish an understanding of how the exercise is a symptom of ED patients’ illness and how the interrelationship between that symptom and other symptoms. This may be focused in the patient’s primary care team, in psychoeducation and in the patients’ individual therapy sessions with the physiotherapist, the psychologist, and the psychiatrist.

Many patients have previously carried out their exercise alone and in rigid and repetitive ways. As an integrated part of the treatment philosophy, we emphasize the facilitation of a social arena to enhance patients’ experience of healthy activities in an enjoyable and noncompetitive atmosphere, in daily activities, body-experience groups, and outdoor activities and exercise groups.

Qualified and enthusiastic staff with specific knowledge of both EDs and exercise are viewed as important, and a defined team is responsible for treatment approaches that range from rest to regular exercise in groups. The team consists of two full-time physiotherapists and a number of nurses who have received formal education in, for example, physical activity and mental health, or are certified climbing instructors, and have taken courses in physical activity (e.g., supervised physical activity) and outdoor life. However, all members of staff are trained to recognize when patients have integrated healthier coping strategies or when there has only been a change in their symptoms, such as when the patient reduces the amount of exercise they take and start to self-harm or when they start to experience challenges during meals. A summary of suggested elements for incorporating exercise into an ED treatment are presented in Table 2.

**Table 2:** Summary of exercise in inpatient treatment of eating disorder patients: a chain of approaches from admission to discharge, and from rest to regular exercise groups

Assessment at admission	Motives and maintenance factors (e.g., weight regulation, improved appearance, avoidance of difficult emotions) Consequences if exercise is restricted or stopped Performance of exercise (e.g., rigid or flexible, joyful, social, or done alone) Awareness of bodily signals (e.g., recognize when hungry or tired) Previous exercise experience (e.g., type, frequency, intensity, duration)
Psychoeducation	Relevant topics Healthy exercise Anatomy and bodily functions Rest and relaxation Balance between exercise, rest, and nutrition Exercise as a symptom of an eating disorder Negative and compulsive attitudes and thoughts
Practical supervision and body-oriented work	Assistance to recognize and develop healthy coping strategies in daily activities (e.g., rest, outdoor walks) Relaxation exercises Enhancement of body awareness (e.g., bodily signals, mindfulness)
Healthy exercise	Facilitate a social and noncompetitive atmosphere for enjoyable physical activities Outdoor activities (e.g., mountain hiking, climbing, horse riding) Regular exercise groups
Exercise groups	Based on basic training principles (variation, adjusted progression, enhancing cardiovascular endurance, muscular strength, and endurance) Individual supervision Good restitution
Practical considerations	All patients: The symptoms, capacity, and needs of the patients during treatment Underweight patients: Loss of muscular tissue, low bone density, and other somatic symptoms Planned weight gain must continue
Personnel	Educated personnel in both eating disorder and exercise/body-oriented therapy

### Assessment

In order to target treatment goals and priorities, we experienced quickly that structured assessments and evaluations of the patients’ attitudes and thoughts towards their exercise were important and useful. Already in 2003, we started to investigate several dimensions of this phenomenon. We wanted to know what kind of exercise the patients had performed, whether they enjoyed their exercise, and their motives for exercising (e.g., health, weight regulation, improved appearance, or to avoid difficult emotions). It was necessary to explore how they would react when their ability to exercise was restricted or stopped in accordance with their treatment program. We also investigated the types of exercises they did, such as whether they were performed in a social setting or whether they were performed alone in a rigid and repetitive way. Additionally, we wanted to understand the patients’ ability to recognize their bodily signals (e.g., feeling tired or hungry) and whether they could use or wanted to use this information to adjust their exercise in a healthy way. Furthermore, we wanted to investigate quantitative elements of the exercise they did prior to admission to the ED unit (i.e., frequency, intensity, and duration). Our structured assessment was extended and we developed the Exercise and Eating Disorders (EED) Questionnaire [18–20]. The EED Questionnaire was the first clinically derived self-report questionnaire to assess attitudes to and thoughts about compulsive exercise among ED patients. We have experienced the use of the EED Questionnaire in our unit as useful for both the staff and the patients, and the information is included in treatment planning and evaluation.

In our unit, the structured assessment is carried out by the physiotherapists, but it is supplemented with information and observations from the other staff. The first interview (at admission) focuses on treatment targets and planning. It is important to discover and differentiate between patients’ motives for exercise, as they may require different treatment approaches. For example, we need to determine whether their motives are mainly connected to regulation of emotions, to body image, to compulsive thoughts or as part of a self-harm problem, or a combination of different motives. Former experience of exercise is investigated too, including the type of exercise and amount (i.e., frequency, intensity, and duration). A second assessment is scheduled before the patient enters Stage 2 of the treatment, to adjust their treatment targets and identify the individual challenges regarding exercise at this transition point. A third assessment is scheduled prior to discharge, to make plans for the future.

### Psychoeducation

Psychoeducation of exercise related topics are viewed as important, and are adjusted to the different stages patients have reached in their treatment and the patients’ rate of progress. Psychoeducation is provided on an individual basis during the treatment sessions and in scheduled groups, as well as in practical situations, when patients may be offered assistance to recognize and develop alternative and healthy coping strategies. Relevant topics may cover basic training principles, what constitutes healthy exercise as well as the prerequisites for exercise to become healthy, anatomy, and bodily functions. Additionally, issues relating to rest and relaxation are given attention in psychoeducation and in scheduled approaches in the unit. To achieve the goal of healthy exercise and a healthy lifestyle, a prerequisite is for patients to achieve a balance between issues relating to rest and relaxation, exercise, and nutrition. Typical examples of negative and compulsive attitudes that are addressed are:

- “I want to have a fit body with defined muscles and without body fat.”
- “I have to perform my exercise every day, otherwise I cannot relax.”
- “If I am interrupted, I must start over again.”
- “Rest and relaxation is not necessary and I do not like it. That makes me lazy and fat, and I do not allow myself to feel good about myself”

### Rest and relaxation

Rest periods (30–60 min) after meals are an obligatory part of the patients’ daily schedule to practice relaxation, to be confident about rest after meals as something natural and not harmful. In our chain of approaches, rest and relaxation is perceived as a starting point for the subsequent approaches to increased exercise. According to the schedule, rest and relaxation is done in a specially designated room with all patients together. The staff are present for support and assistance, and may provide instructions for relaxation exercises. Individual adjustments are possible, and patients may be offered alternative solutions if necessary.

### Body awareness groups and body-oriented therapy

Patients attend weekly body awareness groups, and are offered weekly sessions of individual body-oriented therapy sessions with the physiotherapist (about 45 min). The treatments in the body-oriented therapy include relaxation exercises, body awareness movements, and massages, and are administered by physiotherapists, and nurses from the unit serve as co-therapists. The group sessions last 30 min in Stage 1 of the treatment program and 60 min in Stage 2.

### Outdoor walks

In Stage 1, the most severely ill patients may only be allowed to get some fresh air outside the unit. When their medical state is satisfactory, daily outdoor walks in the area are planned. Initially, the patients take short walks (15 min) during which they are accompanied by the staff. When they make progress in their treatment, they are given more responsibility, such as taking longer walks either alone or accompanied by other patients. The patients are eventually free to plan their walks, as long as the timing does not interfere with the scheduled treatment program.

### Outdoor activities

For patients to experience healthy life and increased bodily function in an enjoyable and social arena, outdoor activities are included as part of the treatment program. One day every week, the patients are exposed to various outdoor activities, such as mountain hiking, climbing and horse riding. The activities are adjusted to the patients capacity and health state. In addition to experiencing the outdoor activities, the patients can practice eating their meals in other surroundings and other places than at the kitchen table as usual.

### Regular exercise groups

In Stage 2 and Stage 3, the patients are allowed to attend two regular exercise groups per week. In the case of underweight AN patients, the medical examination performed by the psychiatrist will determine the patient’s attendance, and whether individual adjustment is necessary due to low bone density, loss of muscular tissue, or other somatic symptoms. One condition of admission to the unit is that the patient’s weight gain continues according to plan for the underweight patients.

The sessions include strength training and aerobic exercise (each session lasts 60 min). The patients are exposed to different levels of exercise intensity, but mainly moderate level. The exercise groups are designed by applying basic training principles, such as variation in the activities, individually supervised and adjusted progression, good restitution, and enhanced general cardiovascular endurance, muscular strength, and endurance.

We do not use watches or measure heart rates, steps, calories, or the amount of fat burned. However, we may include measures of heart rate to increase awareness of bodily signals. There may be a discrepancy between patient’s evaluations of their own bodily state and/or capacity and their bodily function and more objective measures of these.

### Weekend permission and after discharge

During admission, we explore how exercise and physical activity could play a role in the patient’s healthy life after discharge, and provide conditions for the transfer of new experiences. The patients may have homework during their weekend permissions, such as taking part in exercise activities at home together with their family and friends. If necessary, issues relating to exercise may be emphasized as a part of treatment after admission.

### Other issues

In recent years, we have experienced new challenges. In social media it has become common to share detailed information about exercise in open groups and in closed, special interest groups. In our unit, we have the following clause in the treatment agreement: “When admitted, information regarding treatment and symptoms should not be shared on social media.” It is not always easy to monitor the patients in this respect, but we encourage them to cooperate by following the agreement they have signed and focusing on their own recovery. It is also important to investigate these issues together with the patients as a part of developing healthy attitudes to exercise.

With the exception of one patient who broke their arm after a fall, none of our patients have had any negative somatic consequences in connection with our exercise approaches, or any negative psychological consequences. However, difficulties encountered in the management of exercise symptoms due to lack of full cooperation have in some cases led to the premature discharge of patients.

### Case example

From an early age, “Eva” was dedicated to her schoolwork, and she enjoyed participating in sports and other activities with her friends. At the age of 16 years, a number of incidents influenced Eva’s life: she broke up with a boyfriend, and afterwards she had some negative sexual experiences with another boy, for which she felt she herself was to blame. During that period, Eva’s family observed that she ate less, exercised more, looked sad, spent less time with friends, and her school grades fell. For a long time, she did not tell anyone about having problems due to her traumatic experiences. Eva felt exercising was a good coping strategy because she was able to suppress her difficult emotions, and she enjoyed being admired for her fit appearance. However, the exercising gradually became a problem. She was jogging until she felt exhausted, several times every day. If she could not complete a jog, she restricted her meals and did not allow herself to relax.

Eva was persuaded by her parents to seek help, and after some time she was referred to inpatient treatment. She was 20 years of age when she was admitted. Her BMI at admission was 14 (37.2 kg). After the introductory week, Eva was ambivalent, especially towards the restriction of her exercise. However, she agreed to accept active treatment, mainly to satisfy her parents’ wishes. During the first 4 weeks, Eva did not manage to finish all of her meals but, with a lot of support from her primary care team, she gradually made progress. Her body weight increased to 39.3 kg, and the program’s target weight gain of 0.7 kg per week was included in her treatment. It was estimated that Eva would need about 19–20 weeks to reach a BMI of 20 (equivalent to 53.1 kg). On the basis of these calculations, the total length of Eva’s stay was estimated as 30 weeks, including the 6 weeks of stabilization. The reports showed that her medical state was stable, but reduced bone mass and loss of muscular tissue were factors to be aware of when planning attendance at exercise sessions.

The assessment regarding exercise issues revealed that Eva’s main motives for exercising had originally been to avoid feeling depressed and being reminded of her difficult life experiences, but she also wanted to enhance what she considered was an ideal appearance and to burn calories after eating. The nature of Eva’s exercise had thus changed from being enjoyable to being rigid and repetitive. Her ability to notice her bodily signals was reduced (especially feelings of hunger and being tired), and if she recognized such signals, she tried to ignore them. In addition, Eva was convinced that all of her soft tissue was fat, and that muscles should never be relaxed, but should be firm in order to be strong. The unit’s staff evaluated that attitude and thoughts towards compulsive exercise, across several dimensions, was a major symptom of Eva’s ED.

### Stage 1 of the treatment

In Stage 1 of Eva’s treatment program, her care team had to be attentive to addressing her reasons for sustaining her motives for exercising, and to help her to discover and integrate healthy alternatives into her behavior. Her dysfunctional body-related thoughts and emotions were systematically investigated during her daily activities and therapy appointments. Eva needed individual adjustments in connection with resting hours because she did not want to relax and thought that rest would make her a lazy person. She was allowed to rest in the living room rather than in the designated resting room. A lot of support, psychoeducation, and exercises in relaxation were necessary before Eva could use the resting room unattended.

Eva was initially allowed two 15-min walks per day, accompanied by a member of staff. To some extent, she enjoyed the walks because she felt it was easier to talk about her situation while she was walking. However, she also experienced the constraints placed on the walks, such as the timeframe and walking speed, as difficult. Eva regularly tried to persuade the accompanying staff member to make changes, and became angry when her requests were denied. Nevertheless, progress was made, and Eva was able to take more responsibility. To some extent, she gained insights into why her drive for increased activity was due to her compulsive behavior and why she needed affect regulation. These connections between her emotions and exercise were addressed in individual therapeutic sessions.

However, during Stage 1, the cooperation between Eva and her care team was challenging. A couple of weeks before her transition to Stage 2, her planned weight gain stopped. It was reported that she was finishing her meals, but the night nurse wondered whether Eva was exercising in her hospital room late in the evenings. Eva denied such behavior, and although she was offered extra support, no progress was made. Due to the signed treatment agreement, the team decided that Eva should leave the treatment unit for 1 week. When she returned, Eva admitted that she had been exercising in her room. However, she had decided that she wanted to continue treatment and was now willing to cooperate. Following consultations between Eva and her care team, her treatment plans and targets were renewed.

### Stage 2 of the treatment

During Stage 2, Eva made progress and her weight gain continued according to plan. She was able to take responsibility for her outdoor walks. She discovered that she liked to take photos, and she started to do that as an activity during her walks. Eventually, the walking speed and distance became less important to her.

Additionally, Eva was able to attend the weekly exercise groups. It became evident that she faced several challenges, sine she found it difficult to stop pushing herself during workouts, and to adjust to lower and more varied intensity levels. It was therefore necessary for her to practice awareness of bodily signals of being hungry, thirsty, and tired, to distinguish between feeling restless and having energy, and to consider such signals in order to regulate her exercise in a healthy way. Gradually, Eva regained here experience of exercise as enjoyable, and she managed to reduce her comparisons of herself with others. Her challenges related to acceptance of her body image and reaching a target weight equivalent to a BMI of 20. These issues were addressed together with the specialist physiotherapists in individual therapy sessions and body image groups. The relationship between body and emotions was discussed with Eva, and differences between reality and subjective interpretations were explored.

Additionally, Eva took part in the outdoor activities. These activities were a positive part of her recovery process, and she especially enjoyed climbing. She learned to practice social activities that could be part of her life after discharge.

During the admission period, Eva regularly had permission to return home at the weekends. In Stage 3, the permitted times were prolonged, and her attention was directed towards life outside the unit, where she would return to school and to her friends and family. The weekend trips were planned and evaluated each week, and workouts were planned for Eva to practice at home. For example, she could take part in exercise activities together with friends to practice new coping strategies. After discharge, Eva was to continue in outpatient treatment, and appointments with a physiotherapist were part of her outpatient treatment to continue to integrate healthy exercise in her daily life.

## Discussion

In this paper, we have described 15 years of our clinical experience with exercise approaches as an integral part of our inpatients’ treatment programs. We have presented our recommendations for the treatment program, and illuminated our clinical management through a case example. During these 15 years, we have experienced exercise approaches as a beneficial supplement to our treatment program, and we have not experienced any risks or harm to the patients. These experiences are in agreement with those of other researchers [2, 3, 5]. We consider it necessary and important to address and include treatment approaches ranging from admission to discharge, and from rest to exercise, which emphasize a healthy lifestyle to ED inpatients. In addition to our clinical experience, our research findings have not shown any adverse influence on weight gain in underweight patients during treatment [21], as has also been reported by other researchers [2, 3, 5, 14]. Our recommendations have a number of features in common with the proposal presented in 1994 by Beumont and colleagues, that a supervised exercise program for ED patients would be a beneficial supplement to existing treatment, as well as more recent proposals [15, 22], and they may contribute to the process of developing future guidelines in the field of ED.

Although, the results reported in Noetel et al.’s recent paper on clinical experts’ attitudes towards exercise among adolescents may not be directly comparable with an adult population [1], many issues are recognizable. As shown in our recommendations and as illuminated in the case of Eva, we agree with Noetel et al. that *compulsive exercise* is the most precise term to describe the challenges ED patients face with regard to exercise. In addition, we acknowledge that it is more important to address the qualitative elements of exercise than the quantitative elements [7, 12]. Our experience is also in accordance with Meyer and colleagues [7], highlighting regulation of negative emotions, rigidity, perceived negative consequences if exercise is restricted or stopped as necessary to investigate in addition to weight and shape motives.

## Conclusions

Our experience and recommendations support earlier proposals for treatment approaches to exercise and exercise-related issues as a beneficial supplement to the treatment of ED inpatients. We have not experienced any adverse influences on patients’ recovery processes, such as their rate of weight gain. Our intention is that this paper will be a contribution to the field of ED, the integration of exercise approaches in the inpatient treatment of ED and development of clinical guidelines.

## Abbreviations

AN: anorexia nervosa
BED: binge eating disorder
BMI: body mass index
BN: bulimia nervosa
ED: eating disorder
EED: Exercise and Eating Disorder Questionnaire
ICD: International Classification of Diseases
RKSF: Regionalt kompetansesenter for spiseforstyrrelser (Specialist eating disorder unit)
